# Reimagining simulation for quality and safety in healthcare: connecting paradigms, methods, and communities

**DOI:** 10.1186/s41077-025-00397-5

**Published:** 2025-11-30

**Authors:** Victoria Brazil, Susan Eller, Komal Bajaj

**Affiliations:** 1 Bond University, Gold Coast, Australia; 2 Stanford University, Palo Alto, CA USA; 3 Albert Einstein College of Medicine, New York, USA

The healthcare simulation community has embraced its role in improving quality and safety in healthcare. Simulation is an effective tool for the education and training of healthcare professionals as a method to support improvement. This work is impactful and ongoing. More recently, the role of simulation has expanded to directly explore and improve systems, safety challenges, physical spaces, and processes of care within health services, conceptually framed as ‘systems focused’ or translational simulation [1–3]. Exemplar projects that have adopted this conceptual framing have demonstrated substantial improvements in quality outcomes and system performance [4–6]. But despite these demonstrated impacts, translational simulation methodologies are barely perceptible in most healthcare quality and safety practice. We have more work to do.

Healthcare system complexity is rising (digital transformation, new spaces, new treatments, virtual care), but many changes go live untested in the real context of use. Capital projects often require costly rework and downtime that could be avoided by early simulation testing. Crises (pandemics, climate events) require rapid, reliable service reconfiguration under uncertainty. To realise the potential for simulation to address these challenges and more, we need to build upon and extend our aims and our methodologic perspectives. We need thoughtful approaches to simulation design, delivery and debriefing that focus on systems and complexity. We need to embed data strategies into our systems-focused simulation. We need to connect more closely with healthcare improvement methods, communities and theoretical foundations. In this editorial, we articulate the necessity and the urgency of this work and call for scholars to drive the academic conversations that guide and report it.

## How is simulation currently contributing to healthcare quality and safety?

Examples of system-focused simulation to improve healthcare quality (effectiveness, efficiency, safety, timeliness, patient centredness and accessibility) are numerous. Improving time-based targets like ‘door to needle time’ in stroke care [4], or ‘time to CT Scan’ in trauma care [7] are straightforward illustrations; simulation of these patient journeys allows healthcare teams who do the work to find gaps in their system and address them. The COVID-19 pandemic provided further illustration [8–11]; simulation of practices like proning of ICU patients [12] or ‘COVID-safe’ airway management [13] tested processes before use in real patient care. Simulation to support design of physical spaces and clinical equipment is another example: Are the hospital lifts the right size [14]? Is our airway equipment set up optimally for staff to perform procedures [15]? Does the room layout support trauma teams to work ergonomically [16, 17] and free of latent safety threats [18–21]? Simulation has become a tool for healthcare teams to become agents of their own improvement in everyday care, whether improving performance in post-partum haemorrhage [22, 23], improving blood transfusion safety and speed [24], safety of neonatal ICU transfers [25], or myriad other contexts. Although diverse in context, these examples share (1) a direct focus on the processes and systems that affect health service performance, and (2) emerging simulation practices that may differ from those employed for educationally focused simulation.

## How has our simulation practice been adapted for systems focus and improvement?

The purpose of translational simulation activities is the exploration and improvement of systems and processes [2], rather than solely on developing the skills or abilities of individuals or teams. This paradigmatic shift demands new or adapted approaches to simulation design, delivery, debriefing and data strategies [26]. Choice of simulation modality may have different considerations to optimise simplicity and alignment to purpose [27]. In situ simulation – delivery of simulation in real clinical environments – becomes an attractive choice for exploring or improving processes and environments in which care is delivered [20, 28–30], but creates new safety risks to mitigate [31]. Debriefing approaches need to be adapted for the purpose of collecting data on system performance, including perceptions of participants, rather than primarily being a place where healthcare professionals can reflect on their individual or team performance [32, 33]. Our mindset shifts; toward simulation that supports data collection to inform change towards more reliable systems [34, 35]. These new or revised practices might draw upon conceptual foundations beyond learning sciences, including organisational science, systems engineering, implementation science, complex adaptive systems theory and safety science [2].

## How do we build connections and advocate for translational simulation within quality and safety practice?

The existing literature shows that translational simulation programs are variably connected with their quality improvement counterparts in health services and in scholarly conversations [36]. These potential connections include governance arrangements [37], alignment of improvement methods and tools [5, 24], multidisciplinary project teams, faculty development activities [38] and shared academic conversations [39, 40] and reporting guidelines [41]. Connections may include shared mindsets i.e. building on practice and research in debriefing developed by the simulation community over many years, routine *clinical* debriefing of surgical cases has resulted in decreased surgical mortality, improved safety culture, and cost savings [42]. We suggest there is more to do if systems-focused simulation is to realise its full potential for contributing to quality and safety in healthcare, and to be recognised by the healthcare quality improvement community as a practical and feasible approach to incorporate into their practice.

## What might the future look like for healthcare quality improvement that is integrated with and supported by simulation?

To advance the mission of simulation to improve healthcare system and service quality, we argue that we need to build on prior work and dramatically reimagine the intersection of quality improvement and simulation in healthcare. What might that look like? Simulation as a default, routine step in every significant change to care (process, space, technology), not a special event. Collaboration with quality and safety units in health services: shared governance, shared budget, shared dashboards. Patient, family, and community engagement in simulation-based quality improvement. Blended modalities: exploring and testing using tabletop simulation, then in situ scenarios, then digital twins (*virtual replica of a process*,* or system that is continuously updated with real-time data to enable predictive modelling* [43]) as a project pipeline. Ensuring operational readiness for teams moving into new spaces. And led by a recognised workforce: defined roles, competencies, faculty development, and career pathways for translational simulation.

## How can practitioners and scholars contribute?

We seek submissions from simulation and quality improvement (QI) practitioners and scholars to a special collection in Advances in Simulation: *Connecting simulation with healthcare quality and safety*. We invite manuscripts that illustrate one or more ways in which we can build connections in governance, methodologies, data science, operational teams, faculty development or research. Submissions should go beyond a simple shared purpose of improvement and extend our understanding of the nuanced and multifaceted ways that connections may be built. Examples might include: design principles and data strategies for systems-focused simulations (measures, definitions, collection templates); guidance on fusing simulation and clinical debriefing within quality improvement; governance strategies: how to embed sim within clinical risk, capital projects, and procurement sign-offs; healthcare logistics, i.e. simulations for inventory management, device roll-outs, and contingency planning (e.g., shortages, recalls); faculty development curricula for translational simulation practitioners and QI teams learning each other’s tools; digital integration - simulation testing of EHR-linked triggers and workflows; data pipelines that move simulation findings directly into QI dashboards/statistical process control; and open repositories - scenarios, debrief guides, measurement tools, and sample dashboards.

Translational simulation offers us a test bench for organisational learning. It is a practical strategy for exploring and improving healthcare before, during and after we embed practice. We invite submissions that make this bridge visible - methods, governance, and outcomes that show how simulation and quality improvement together might deliver reliable change at scale.

## Data Availability

Not applicable.
